# Association of body composition with odds of breast cancer by molecular subtype: analysis of the Mechanisms for Established and Novel Risk Factors for Breast Cancer in Nigerian Women (MEND) study

**DOI:** 10.1186/s12885-021-08775-8

**Published:** 2021-09-25

**Authors:** Tomi Akinyemiju, Kelley Jones, Anjali Gupta, Taofik Oyekunle, Veeral Saraiya, April Deveaux, Omolola Salako, Allison Hall, Olusegun Alatise, Gabriel Ogun, Adewale Adeniyi, Omobolaji Ayandipo, Thomas Olajide, Olalekan Olasehinde, Olukayode Arowolo, Adewale Adisa, Oludolapo Afuwape, Aralola Olusanya, Aderemi Adegoke, Trygve O. Tollefsbol, Donna Arnett, H3 Africa Kidney Research Network, Samuel Ajayi, Yemi Raji, Timothy Olanrewaju, Charlotte Osafo, Ifeoma Ulasi, Adanze Asinobi, Cheryl A. Winkler, David Burke, Fatiu Arogundade, Ivy Ekem, Jacob Plange-Rhule, Manmak Mamven, Michael Mate-kole, Olukemi Amodu, Richard Cooper, Sampson Antwi, Adebowale Adeyemo, Titilayo Ilori, Victoria Adabayeri, Alexander Nyarko, Anita Ghansah, Ernestine Kubi Amos-Abanyie, Priscilla Abena Akyaw, Paul L. Kimmel, Babatunde L. Salako, Rulan S. Parekh, Bamidele Tayo, Rasheed Gbadegesin, Michael Boehnke, Robert Lyons, Frank Chip Brosius, Daniel Clauw, Chijioke Adindu, Clement Bewaji, Elliot Koranteng Tannor, Perditer Okyere, Chuba Ijoma, Nicki Tiffin, Junaid Gamiedien, Friedhelm Hildebrandt, Charles Odenigbo, Nonyelun Jisieike-Onuigbo, Ifeoma Modebe, Aliyu Abdu, Patience Obiagwu, Ogochukwu Okoye, Adaobi Solarin, Toyin Amira, Christopher Esezobor, Muhammad Makusidi, Santosh Saraf, Victor Gordeuk, Gloria Ashuntangtang, Georgette Guenkam, Folefack Kazi, Olanrewaju Adedoyin, Mignon McCullough, Peter Nourse, Uche Okafor, Emmanuel Anigilaje, Patrick Ikpebe, Tola Odetunde, Ngozi Mbanefo, Wasiu Olowu, Paulina Tindana, Olubenga Awobusuyi, Olugbenga Ogedegbe, Opeyemi Olabisi, Karl Skorecki, Ademola Adebowale, Matthias Kretzler, Jeffrey Hodgin, Dwomoa Adu, Akinlolu Ojo, Vincent Boima, Adetola Daramola

**Affiliations:** 1 Department of Population Health Sciences, School of Medicine, Duke University, Durham, NC USA; 2 Duke Cancer Institute, School of Medicine, Duke University, Durham, NC USA; 3 Duke Global Health Institute, Duke University, Durham, NC USA; 4 Trinity College of Arts and Sciences, Duke University, Durham, NC USA; 5 Department of Epidemiology, University of North Carolina Gillings School of Global Public Health, Chapel Hill, NC USA; 6 College of Medicine & Lagos University Teaching Hospital, University of Lagos, Lagos, Lagos State Nigeria; 7 Department of Pathology, School of Medicine, Duke University, Durham, NC USA; 8 Obafemi Awolowo University Teaching Hospital, Ile-Ife, Osun State Nigeria; 9 University College Hospital, University of Ibadan, Ibadan, Oyo State Nigeria; 10 Federal Medical Center, Abeokuta, Ogun State Nigeria; 11 Our Lady of Apostle Catholic Hospital, Ibadan, Oyo State Nigeria; 12 University of Alabama at Birmingham, Birmingham, AL USA; 13 University of Kentucky, Lexington, KY USA; 14 University of Kansas Medical Center, Kansas City, KS USA

**Keywords:** Body composition, BMI, Breast cancer, Nigeria, Molecular subtype, Hormone receptor

## Abstract

**Background:**

The association between obesity and breast cancer (BC) has been extensively studied among US, European and Asian study populations, with often conflicting evidence. However, despite the increasing prevalence of obesity and associated conditions in Africa, the continent with the highest age-standardized BC mortality rate globally, few studies have evaluated this association, and none has examined in relation to molecular subtypes among African women. The current analysis examines the association between body composition, defined by body mass index (BMI), height, and weight, and BC by molecular subtype among African women.

**Methods:**

We estimated odds ratios (ORs) and 95% confidence intervals (95% CI) for the association between measures of body composition and BC and molecular subtypes among 419 histologically confirmed cases of BC and 286 healthy controls from the Mechanisms for Established and Novel Risk Factors for Breast Cancer in Women of Nigerian Descent (MEND) case-control study.

**Results:**

Higher BMI (aOR: 0.79; 95% CI: 0.67, 0.95) and weight (aOR: 0.83; 95% CI: 0.69, 0.98) were associated with reduced odds of BC in adjusted models, while height was associated with non-statistically significant increased odds of BC (aOR: 1.07, 95% CI: 0.90, 1.28). In pre/peri-menopausal, but not post-menopausal women, both higher BMI and weight were significantly associated with reduced odds of BC. Further, higher BMI was associated with reduced odds of Luminal A, Luminal B, and HER2-enriched BC among pre/peri-menopausal women, and reduced odds of triple-negative BC among post-menopausal women.

**Conclusions:**

Higher BMI and weight were associated with reduced odds of BC overall and by molecular subtype among West African women. Larger studies of women of African descent are needed to definitively characterize these associations and inform cancer prevention strategies.

## Background

Breast cancer (BC) poses a significant global challenge, with an estimated 2.1 million new cases and over 0.6 million deaths occurring in 2018 [1]. The African continent has the highest age-standardized BC mortality rate globally, with Nigeria, the most populous African nation, experiencing the highest rate within the continent [2]. In addition, the past few decades have been characterized by rapid increases in BC incidence rates among African women [3]. This increase is thought to be partly attributable to an epidemiological transition—a trend characterized by a decline in infectious diseases and an increase in non-communicable diseases, including obesity, obesity-associated conditions, as well as cancer [4, 5]. Women of African descent are also more likely to experience disproportionately high rates of aggressive BC tumors with poorer prognosis [6–8]. Specifically, women of African descent, including African-American women in the United States [9] and Nigerian women, are more likely to be diagnosed with BC over-represented by the triple-negative (TN) subtypes, characterized by estrogen (ER), progesterone (PR), and human epidermal growth factor-2 (HER2) receptor negativity [7, 8].

Non-communicable disease risk factors, such as high body mass index (BMI), have been shown to influence BC risk. Several studies have found that higher BMI, an indicator of excess adiposity, is associated with increased risk of post-menopausal BC, but reduced risk of pre-menopausal BC [10, 11]. This association has been observed among diverse populations, including European [12], Latin American [13], and Asian [14] women. However, the few studies in Nigeria, where obesity rates are rapidly increasing [15], are mixed. Two studies have noted no significant associations between BMI and BC risk [16, 17], while studies assessing other measures of excess adiposity, such as waist circumference and waist-to-hip ratio, observed an association with increased risk of BC in Nigerian women [17, 18]. These measures of excess adiposity have been associated with metabolic dysregulation, including insulin resistance and hyperlipidemia [19], which have separately been shown to increase BC risk [20]. Additionally, studies considering body height have found a positive association between height and BC risk [21, 22], including two studies among Nigerian women [16, 23]. Molecular BC subtype has been shown to modify the association between excess adiposity and BC risk. Obese women were found to have a higher risk of TN and Luminal A BC than normal-weight women, whereas normal-weight women were more likely to present with HER2+ BC in a population from the United States [24]. Another United States study found that elevated waist-to-hip ratio was associated with increased risk of Luminal A BC among post-menopausal women, and increased risk of basal-like (TN) BC in both pre- and post-menopausal women [25]. However, no study to date has examined the link between BMI and BC molecular subtypes in West African women.

Improved understanding of the impact of obesity on BC and molecular subtypes in West African women will add to the growing literature that can help inform locally relevant BC prevention strategies and contribute to enhancing existing risk prediction and prognostic models. Findings will also contribute to improved understanding of the biological mechanisms underlying aggressive tumor subtypes in women of African descent. The purpose of the current study was to evaluate the association between BMI, height, and weight with odds of BC among West African women, and to examine differences across molecular subtypes.

## Methods

### Study design

**Fig. 1:**
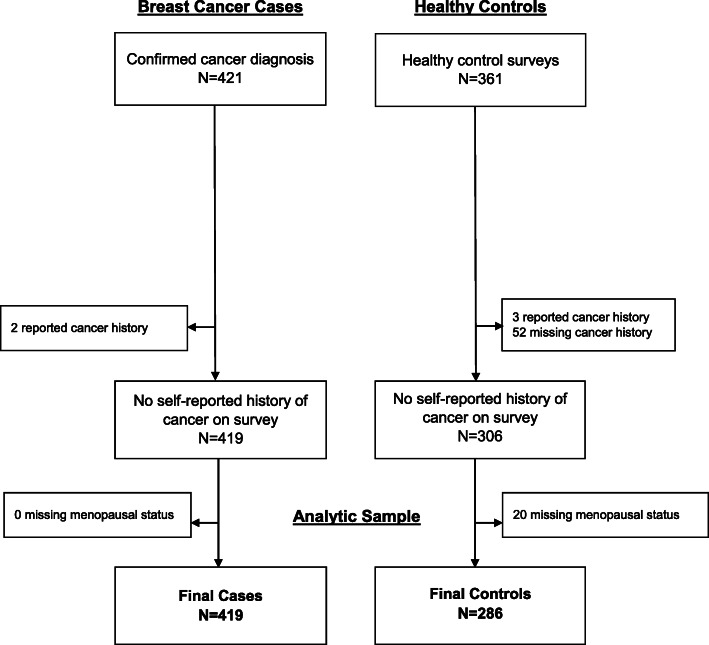
CONSORT diagram for MEND body composition analysis

The Mechanisms for Established and Novel Risk Factors for Breast Cancer in Women of Nigerian Descent (MEND) study has been previously described in detail [26]. Briefly, newly diagnosed BC patients from four tertiary hospitals in southwestern Nigeria were recruited into the research study. A trained research nurse at each site introduced the study and explained study requirements in detail to potential participants during clinical visits, and interested participants were assessed for eligibility. Exclusion criteria included other medical conditions that could interfere with participation in the study, mental impairment, or not being able to communicate in English themselves or through a family member to complete the survey. Written and verbal informed consent was obtained from all study participants. A comprehensive study questionnaire was administered to obtain information on sociodemographic characteristics, reproductive history, and self and family history of cancer. Anthropometric measurements were obtained by the research nurse, followed by blood sample collection, and tumor and adjacent normal tissue biopsy sample collection. All biospecimen were collected prior to any chemotherapy or surgical treatment. Tissue samples were processed at the clinic and stored in − 80 °C freezers until shipment to the United States for further analysis. Participants received a N500 telephone recharge card (approximately US $1.50) as well as supplies needed for their clinical biopsy (biopsy needle and ancillary items). Data on healthy controls without BC were obtained from the Human Heredity and Health Africa (H3A) Chronic Kidney Disease (CKD) case-control study; methodology for the H3A CKD study has been described elsewhere [27]. Briefly, H3A recruitment occurred between 2015 and 2017, overlapping with case recruitment and included the recruitment of healthy, community-based adult women in southwestern Nigeria and Ghana. Extensive socio-demographic, clinical, family history and behavioral risk factor data was collected from all control participants; those with history of cancer or missing data on cancer history were excluded from analysis. Overall, 419 BC cases and 286 healthy controls were included in the current analysis (Fig. 1). All study procedures were approved by Duke University and the participating hospitals’ Institutional Review Boards.

### Breast cancer cases and subtyping

BC diagnosis was confirmed in one of two ways: 1) pathology reports based on clinical tumor biopsy samples from the diagnosing hospital in Nigeria and reviewed by a trained pathologist, or 2) research tumor biopsy samples collected at recruitment (same time as clinical biopsy) and shipped to the United States for review by a pathologist. Participants were considered a confirmed cancer case if either report indicated a cancer diagnosis. Confirmed BC tumor samples were subjected to immunohistochemistry (IHC) either at the diagnosing hospital in Nigeria following standard protocol, or at the Duke University BioRepository and Precision Pathology Center. When both Nigeria and Duke IHC results were available, the Duke typing was used because it constituted the majority of IHC data on cases. Estrogen receptor and progesterone receptor status were scored using the Allred method [28, 29]. The proportion of nuclear positivity was scored as 0 (0%), 1 (< 1%), 2 (1–10%), 3 (11–33%), 4 (34–66%) or 5 (67–100%); intensity of the staining was scored as 0 (none), 1 (mild), 2 (moderate), or 3 (strong). These two scores were summed into positive (3–8) or negative (0–2). HER2 was scored as negative (scores 0, 1), equivocal (score = 2), positive (score = 3) [30]. Cancer subtype was then classified as Luminal A (ER+ and/or PR+ / HER2-), Luminal B (ER+ and/or PR+ / HER2+), TN (ER−/PR−/HER2-), or HER2 (ER−/PR−/HER2+). In total, 169 cases had available data on ER/PR/HER2 status for molecular subtype classification.

### Measures

Anthropometric measurements for cases and controls were collected by trained research staff at enrollment, and included height, weight, and blood pressure. BMI was calculated from height and weight as kg/m^2^. BMI, height, and weight were categorized into quartiles as well as continuous standardized variables by subtracting the sample mean and dividing by the sample standard deviation (SD). Quartiles were used rather than World Health Organization BMI categories (< 18.5 kg/m^2^ underweight, 18.5 - < 25 normal weight, 25 - < 30 overweight, and 30+ obese) because it has been documented that these categories may not accurately capture risk in populations of African descent [31]. Reproductive and clinical history were self-reported by participants, and study covariates included age at menarche, parity, gravidity, menopausal status, and prior hypertension diagnosis. Participants also self-reported whether they had a history of other types of cancer; those with a positive history of cancer (< 1% of cases and controls) or missing personal cancer history data (15% of controls) were excluded from the analysis. Missing data for all measures were tabulated by case/control status. Variables with < 10% missing for both cases and controls were replaced with the median or modal value of the cases or controls, respectively, while variables with > 10% missing were not imputed.

### Analytical approach

Descriptive statistics of proportions and frequencies for categorical variables and means (SD) or medians (first quartile, third quartile) for continuous variables were used to characterize the sample by case/control status and BMI quartile. Differences in characteristics were tested using χ^2^ tests for categorical variables and Wilcoxon rank-sum or Kruskal-Wallis nonparametric tests for continuous variables. The distribution of BMI was evaluated by case/control status and stratified by menopausal status and age group, respectively. The associations between body composition measures (BMI, height, and weight) and BC were tested using logistic regression models. Each body composition measure was assessed separately in a series of models: unadjusted, adjusted for age only, and adjusted for age, age at menarche, number of pregnancies, number of births, menopausal status, and prior hypertension diagnosis. A final model adjusted for BMI, height, and weight simultaneously in addition to all previous covariates. The primary models assessed quartiles as a categorical variable, with the lowest quartile set as the reference group. To test for trend across quartiles, the main exposure was analyzed as a continuous variable [1–4]. Finally, the standardized values for BMI, height, and weight were examined to determine the association per unit standard deviation (SD) increase in the measure with the odds of being a cancer case. These models were repeated with stratification for menopausal status. The subset of cases with molecular subtyping was analyzed using multinomial logistic regression models, specifying control status as the outcome reference group. The previous models were repeated to assess the odds of Luminal A, Luminal B, TN, or HER2 cancer subtypes. We assessed whether H3A control participants recruited from Nigeria and Ghana were significantly different in relation to key body composition measures; there were no significant difference between the groups, and in sensitivity analysis examining the analytical models using Nigerian controls only, results from the overall analysis were consistent, therefore analysis with the entire cohort of controls is presented. SAS v9.4 (SAS Institute, Cary, NC) was used for all analyses and significance was set at α = 0.05.

## Results

**Fig. 2:**
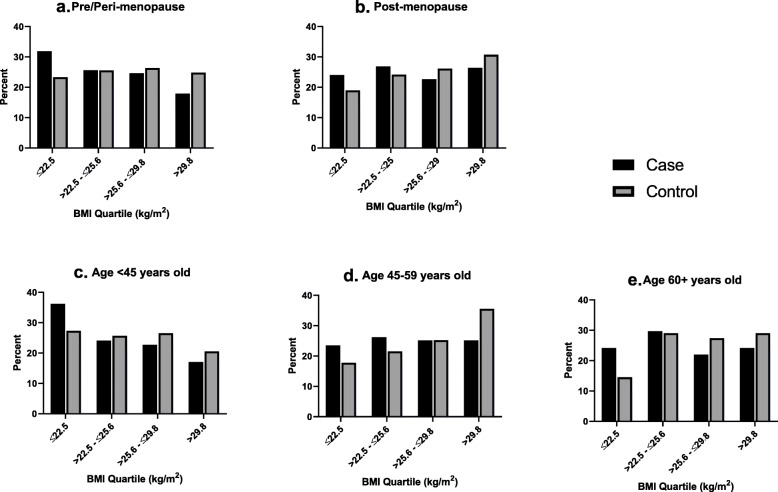
BMI quartile by case/control status and clinical factors. **a** Distribution of BMI quartiles by case/control status among pre/peri-menopausal participants. **b** Distribution of BMI quartiles by case/control status among post-menopausal participants. **c** Distribution of BMI quartiles by case/control status among participants younger than 45 years old. **d** Distribution of BMI quartiles by case/control status among participants aged 45–59 years old. **e** Distribution of BMI quartiles by case/control status among participants 60 years or older

A total of 419 BC cases and 286 healthy controls were included in the analysis (Fig. 1). Median age at diagnosis or enrollment (48 years vs. 47 years), median age at menarche (15 years vs. 15 years), number of pregnancies (5 vs. 4) and number of births (4 vs. 4) was similar among cases and controls (Table 1). However, cases were less likely to report any prior use of hormone replacement therapy compared with controls. Approximately half (54%) of cases and 60% of controls were overweight or obese, and controls were more likely to report a prior diagnosis of diabetes or hypertension. Among cases, the most common subtype was triple-negative (31%), and 38% of cases were diagnosed at advanced grade (grade 3). Across BMI quartiles (Table 2), those in the highest quartile were older (*p* < 0.001), more likely to have a prior diagnosis of hypertension (*p* < 0.001), report ever use of HRT (*p* = 0.009) and be ever pregnant (*p* = 0.01). Furthermore, the distribution of BMI by case/control status stratified by menopausal status and age group (Fig. 2) indicated that the proportion of cases compared to controls in the lowest BMI quartile was higher across categories of menopausal status and age.

**Table 1:** Clinical and reproductive characteristics among breast cancer cases and controls

Study characteristics	Case *N* = 419	Control *N* = 286
Demographics		
Age (years)^a^	48.0 (42.0, 58.0)	47.0 (40.0, 58.0)
Clinical characteristics		
BMI category		
Underweight (< 18.5)	21 (5.0%)	8 (2.8%)
Normal weight (18.5 - < 25)	172 (41.1%)	106 (37.1%)
Overweight (25 - < 30)	136 (32.5%)	94 (32.9%)
Obese (30+)	90 (21.5%)	78 (27.3%)
BMI quartile		
≤ 22.5	117 (27.9%)	60 (21.0%)
> 22.5 - ≤25.6	110 (26.3%)	71 (24.8%)
> 25.6 - ≤29.8	99 (23.6%)	75 (26.2%)
> 29.8	93 (22.2%)	80 (28.0%)
Height, cm ^a^	160.0 (156.0, 164.1)	159.7 (155.0, 163.5)
Weight, kg ^a^	64.9 (55.3, 75.8)	66.0 (58.0, 78.0)
High blood pressure at enrollment	134 (32.0%)	95 (33.2%)
Systolic BP ^a^	126.0 (114.3, 142.3)	128.5 (115.3, 144.0)
Diastolic BP ^a^	80.0 (70.7, 88.7)	77.0 (70.0, 87.7)
Prior diabetes diagnosis		
Yes	6 (1.4)	43 (15.0)
No	411 (98.1)	190 (66.4)
Missing	2 (0.5)	53 (18.5)
Prior hypertension diagnosis	82 (19.6%)	134 (46.9%)
Reproductive history		
Age at menarche ^a^	15.0 (14.0, 16.0)	15.0 (14.0, 17.0)
Ever pregnant	399 (95.2%)	269 (94.1%)
Number of pregnancies ^a,b^	5.0 (3.0, 6.0)	4.0 (3.0, 6.0)
Number of births ^a,b^	4.0 (3.0, 5.0)	4.0 (2.0, 5.0)
Menopausal status		
Pre- or peri-menopause	207 (49.4%)	133 (46.5%)
Post-menopause	212 (50.6%)	153 (53.5%)
Ever used HRT		
Yes	3 (0.7)	41 (14.3)
No	416 (99.3)	189 (66.1)
Missing	0 (0.0)	56 (19.6)
Cancer type	*(n = 169)*	n/a^c^
Luminal A	46 (27.2%)	
Luminal B	34 (20.1%)	
Triple negative	52 (30.8%)	
HER2	37 (21.9%)	
Grade		n/a^c^
1	6 (1.4%)	
2	132 (31.5%)	
3	84 (20.1%)	
Unknown/Missing	197 (47.0%)	
Prior Mammography Screenings	78 (18.6%)	n/a^d^
Any Family History of Cancer	21 (5.0%)	n/a^d^

^a^Median, (Q1, Q3)

^b^Among those who were ever pregnant

^c^Cancer variables are not applicable to control participants

^d^Unavailable for control participants

**Table 2:** Clinical and reproductive characteristics by BMI quartile among breast cancer cases and controls

Study characteristics	1st quartile: BMI ≤ 22.5 *N* = 177	2nd quartile: BMI > 22.5 - ≤ 25.6 *N* = 181	3rd quartile: BMI > 25.6 - ≤ 29.8 *N* = 174	4th quartile: BMI > 29.8 *N* = 173	*P*-value
Case status					.10
Case	117 (66.1%)	110 (60.8%)	99 (56.9%)	93 (53.8%)	
Control	60 (33.9%)	71 (39.2%)	75 (43.1%)	80 (46.2%)	
Demographics					
Age (years)^a^	46.0 (37.0, 55.0)	48.0 (40.0, 58.0)	47.5 (41.0, 56.0)	52.0 (44.0, 59.0)	< .001
Clinical characteristics					
Height, cm ^a^	161.0 (156.0, 164.5)	160.0 (156.0, 164.0)	160.0 (155.0, 163.8)	160.0 (156.0, 164.1)	.69
Weight, kg ^a^	52.0 (48.5, 55.0)	62.0 (58.0, 64.9)	70.9 (66.0, 74.8)	85.9 (80.0, 94.0)	< .001
High blood pressure at enrollment	32 (18.1)	68 (37.6)	65 (37.4)	64 (37.0)	< .001
Systolic BP ^a^	121.7 (109.3, 132.0)	127.0 (114.7, 145.7)	129.0 (116.7, 147.7)	132.0 (119.7, 148.7)	< .001
Diastolic BP ^a^	76.0 (66.7, 84.0)	78.3 (68.7, 89.3)	79.2 (72.3, 88.3)	80.3 (74.7, 89.7)	< .001
Prior diabetes diagnosis					.21
Yes	7 (4.0%)	15 (8.3%)	11 (6.3%)	16 (9.3%)	
No	159 (89.8%)	156 (86.2%)	143 (82.2%)	143 (82.7%)	
Missing	11 (6.2%)	10 (5.5%)	20 (11.5%)	14 (8.1%)	
Prior hypertension diagnosis	32 (21.6%)	41 (28.5%)	55 (37.2%)	65 (48.1%)	< .001
Reproductive history					
Age at menarche ^a^	15.0 (14.0, 16.0)	15.0 (14.0, 16.0)	15.0 (14.0, 16.0)	15.0 (14.0, 17.0)	.54
Ever pregnant	161 (91.0%)	170 (93.9)	166 (95.4)	171 (98.8)	.01
Number of pregnancies ^a,b^	4.0 (3.0, 6.0)	4.0 (3.0, 6.0)	4.0 (3.0, 6.0)	5.0 (4.0, 6.0)	.15
Number of births ^a,b^	3.0 (2.0, 5.0)	4.0 (3.0, 5.0)	4.0 (2.0, 5.0)	4.0 (3.0, 5.0)	.13
Menopausal status					.06
Pre- or peri-menopause	97 (54.8%)	87 (48.1%)	86 (49.4%)	70 (40.5%)	
Post-menopause	80 (45.2%)	94 (51.9%)	88 (50.6%)	103 (59.5%)	
Ever used HRT					.009
Yes	4 (2.3%)	10 (5.5%)	11 (6.3%)	19 (11.0%)	
No	159 (89.8%)	153 (84.5%)	152 (87.4%)	141 (81.5%)	
Missing	14 (7.9%)	18 (9.9%)	11 (6.3%)	13 (7.5%)	

Where applicable, missing values were not used to generate the *p*-value

^a^Median, (Q1, Q3)

^b^Among those who were ever pregnant

**Fig. 3:**
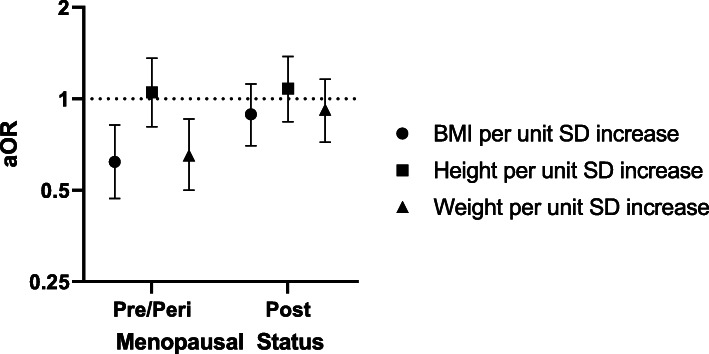
Associations between body composition and breast cancer by menopausal status. Logistic regression models predicting odds of breast cancer. Adjusted for age, age at menarche, number of pregnancies, number of births, and prior hypertension diagnosis

In multivariable regression models (Table 3), BMI in the highest versus lowest quartile (BMI > 29.8 kg/m^2^ vs. < 22.5 kg/m^2^) was associated with reduced odds of BC in adjusted models (aOR: 0.56; 95% CI: 0.33, 0.92; p-trend: 0.02). However, after additionally adjusting for height and weight, the estimate was no longer statistically significant (aOR: 0.32, 95% CI: 0.10, 1.09; p-trend 0.06). Each SD increase in BMI was significantly associated with 21% reduced odds of BC (aOR: 0.79; 95% CI: 0.67, 0.95) in adjusted models but was no longer significant in models additionally adjusting for height and weight (aOR: 0.75, 95% CI: 0.15, 3.72). Weight (kg) in the highest quartile was associated with 37% reduced odds of BC (aOR: 0.63, 95% CI: 0.38, 1.04) in adjusted models, however this was not statistically significant, although each SD increase in weight was associated with significantly reduced odds of BC (aOR: 0.83; 95% CI: 0.69, 0.98). Increased height was not significantly associated with odds of BC in fully adjusted models or in weight and height adjusted models. In sub-group analyses stratified by menopausal status, BMI and weight were significantly associated with reduced odds of BC only among pre/peri-menopausal women (Fig. 3).

**Table 3:** Associations between body composition and breast cancer

	Model 1^a^ OR (95% CI)	Model 2^b^ aOR (95% CI)	Model 3^c^ aOR (95% CI)	Model 4^d^ aOR (95% CI)
**BMI (kg/m**^**2**^**) quartile**				
≤ 22.5	Ref.	Ref.	Ref.	Ref.
> 22.5 - ≤25.6	0.79 (0.52, 1.22)	0.76 (0.49, 1.18)	0.73 (0.44, 1.22)	0.60 (0.31, 1.19)
> 25.6 - ≤29.8	0.68 (0.44, 1.04)	0.65 (0.42, 1.01)	0.63 (0.38, 1.04)	**0.38 (0.15, 0.95)**
> 29.8	**0.60 (0.39, 0.92)**	**0.56 (0.36, 0.86)**	**0.56 (0.33, 0.92)**	0.32 (0.10, 1.09)
*P-trend*	***.01***	***.01***	***.02***	*.06*
aOR (95% CI) per unit SD	**0.81 (0.70, 0.94)**	**0.79 (0.68, 0.93)**	**0.79 (0.67, 0.95)**	0.75 (0.15, 3.72)
**Height (cm) quartile**				
≤ 156.0	Ref.	Ref.	Ref.	Ref.
> 156.0 - ≤160.0	1.05 (0.67, 1.64)	1.07 (0.68, 1.68)	1.05 (0.62, 1.76)	0.90 (0.51, 1.57)
> 160.0 - ≤164.0	1.41 (0.94, 2.10)	1.45 (0.97, 2.16)	1.29 (0.81, 2.05)	1.12 (0.66, 1.90)
> 164.0	1.22 (0.81, 1.84)	1.27 (0.84, 1.91)	1.08 (0.67, 1.73)	0.87 (0.48, 1.60)
*P-trend*	*.17*	*.13*	*.55*	*.85*
aOR (95% CI) per unit SD	1.15 (0.99, 1.34)	1.16 (1.00, 1.36)	1.07 (0.90, 1.28)	1.05 (0.56, 1.96)
**Weight (kg) quartile**				
≤ 56.7	Ref.	Ref.	Ref.	Ref.
> 56.7 - ≤65.0	0.85 (0.56, 1.31)	0.84 (0.54, 1.28)	0.75 (0.46, 1.24)	1.09 (0.55, 2.15)
> 65.0 - ≤77.0	0.89 (0.58, 1.37)	0.87 (0.56, 1.34)	0.93 (0.56, 1.57)	2.04 (0.78, 5.29)
> 77.0	0.74 (0.48, 1.14)	0.71 (0.46, 1.10)	0.63 (0.38, 1.04)	1.70 (0.48, 5.98)
*P-trend*	*.22*	*.16*	*.14*	*.31*
aOR (95% CI) per unit SD	**0.86 (0.74, 1.00)**	**0.85 (0.73, 0.99)**	**0.83 (0.69, 0.98)**	1.06 (0.20, 5.75)

P-trend based on continuous predictors; aOR per unit SD modeled as a one-unit increase in standard deviation of the variable from its mean-centered value

Bolded values indicate significance at *p* < .05

*Abbreviations*: *OR* odds ratio, *CI* confidence interval, *aOR* adjusted odds ratio, *SD* standard deviation

Logistic regression models predicted odds of breast cancer. ^a^Model 1, unadjusted; ^b^Model 2, adjusted for age; ^c^Model 3, additionally adjusted for clinical and reproductive characteristics: age at menarche, number of pregnancies, number of births, menopausal status, and prior hypertension diagnosis; ^d^Model 4, additionally adjusted for all body composition measures: BMI, height, and weight

In multinomial logistic regression models evaluating BC molecular subtypes compared with controls (Table 4), highest versus lowest quartiles of BMI was associated with reduced but not statistically significant odds of each BC subtype, although the association of each SD increase in BMI with reduced odds of Luminal B subtype was statistically significant (aOR: 0.63; 95% CI: 0.42, 0.95). When we stratified by menopausal status, each unit SD increase in BMI was significantly associated with reduced odds of Luminal A (aOR 0.59; 95% CI: 0.35, 0.99), Luminal B (aOR: 0.48; 95% CI: 0.26, 0.90), and HER2-enriched (aOR: 0.49; 95% CI: 0.27, 0.90) BC among pre/peri-menopausal women, and reduced odds of TNBC (aOR: 0.55; 95% CI: 0.32, 0.95) among post-menopausal women. Height in the highest quartile was associated with 78% reduced odds of HER2-enriched BC (aOR: 0.22; 95% CI: 0.06, 0.83). Additionally, each unit SD increase in weight was significantly associated with 55% reduced odds of HER2-enriched BC (aOR: 0.45; 95% CI: 0.24, 0.84) only among pre/per-menopausal women.

**Table 4:** Associations between body composition measures and breast cancer subtype

Model 3	Luminal A aOR (95% CI)	Luminal B aOR (95% CI)	Triple Negative aOR (95% CI)	HER2 aOR (95% CI)
**BMI (kg/m**^**2**^**) quartile**				
≤ 22.5	Ref	Ref	Ref	Ref
22.5–25.5	0.55 (0.22, 1.41)	0.94 (0.36, 2.50)	0.41 (0.16, 1.06)	0.97 (0.34, 2.76)
25.6–29.8	**0.33 (0.12, 0.90)**	0.63 (0.22, 1.76)	0.47 (0.19, 1.14)	0.70 (0.24, 2.07)
> 29.8	0.51 (0.21, 1.24)	0.35 (0.11, 1.13)	0.48 (0.20, 1.13)	0.68 (0.23, 1.97)
*P-trend*	*0.10*	*0.06*	*0.13*	*.38*
aOR (95% CI) per unit SD	0.82 (0.59, 1.16)	**0.63 (0.42, 0.95)**	0.81 (0.58, 1.12)	0.74 (0.50, 1.09)
Pre/peri-menopausal	**0.59 (0.35, 0.99)**	**0.48 (0.26, 0.90)**	0.89 (0.56, 1.42)	**0.49 (0.27, 0.90)**
Post-menopausal	1.03 (0.64, 1.68)	0.66 (0.36, 1.21)	**0.55 (0.32, 0.95)**	1.10 (0.61, 1.98)
**Height (cm) quartile (all)**				
≤ 156.0	Ref	Ref	Ref	Ref
> 156.0 - ≤160.0	0.88 (0.29, 2.68)	0.76 (0.24, 2.41)	1.33 (0.53, 3.36)	0.83 (0.31, 2.22)
> 160.0 - ≤164.0	1.42 (0.58, 3.50)	0.80 (0.29, 2.18)	1.02 (0.43, 2.41)	0.66 (0.27, 1.65)
> 164.0	1.39 (0.55, 3.51)	1.18 (0.46, 3.03)	1.01 (0.41, 2.45)	**0.22 (0.06, 0.83)**
*P-trend*	*.35*	*.74*	*.89*	***.03***
aOR (95% CI) per unit SD	1.20 (0.86, 1.67)	1.09 (0.76, 1.57)	1.05 (0.77, 1.44)	0.74 (0.51, 1.07)
Pre/peri-menopausal	1.23 (0.78, 1.93)	1.39 (0.85, 2.27)	0.77 (0.48, 1.23)	0.72 (0.44, 1.16)
Post-menopausal	1.19 (0.71, 2.01)	0.78 (0.46, 1.33)	1.43 (0.89, 2.28)	0.78 (0.42, 1.44)
**Weight (kg) quartile (all)**				
≤ 56.7	Ref	Ref	Ref	Ref
> 56.7 - ≤65.0	0.46 (0.16, 1.29)	2.14 (0.76, 6.03)	0.52 (0.21, 1.30)	0.84 (0.32, 2.22)
> 65.0 - ≤77.0	0.65 (0.25, 1.73)	1.32 (0.42, 4.18)	0.77 (0.32, 1.88)	0.48 (0.15, 1.54)
> 77.0	0.73 (0.30, 1.75)	0.46 (0.12, 1.77)	0.54 (0.23, 1.30)	0.52 (0.19, 1.44)
*P-trend*	*.67*	*.14*	*.29*	*.15*
aOR (95% CI) per unit SD	0.91 (0.65, 1.27)	0.68 (0.45, 1.01)	0.83 (0.60, 1.14)	0.68 (0.46, 1.01)
Pre/peri-menopausal	0.70 (0.43, 1.16)	0.59 (0.33, 1.06)	0.81 (0.50, 1.30)	**0.45 (0.24, 0.84)**
Post-menopausal	1.10 (0.67, 1.78)	0.62 (0.34, 1.15)	0.68 (0.41, 1.13)	0.99 (0.55, 1.78)

Multinomial logistic regression models predicting odds of breast cancer subtype versus controls. Adjusted for reproductive and clinical characteristics: age, age at menarche, number of pregnancies, number of births, menopausal status (not included in stratified models), and prior hypertension diagnosis

P-trend based on continuous predictors

aOR per unit SD was modeled as a one-unit increase in standard deviation of the body composition variable from its mean-centered value. These models were stratified by menopausal status

Bolded values indicate significance at *p* < .05

*Abbreviations*: *aOR* adjusted odds ratio, *CI* confidence interval, *SD* standard deviation

## Discussion

In the first contemporary cohort of newly diagnosed African BC cases and healthy controls, increasing BMI and weight were significantly associated with reduced odds of BC overall, but no statistically significant association was noted with respect to height. Similar associations were observed among both pre/peri-menopausal and post-menopausal women, but only reached statistical significance among pre/peri-menopausal women. In our analysis of BC subtypes, higher BMI was significantly associated with reduced odds of Luminal A, Luminal B, and HER2-enriched BC among pre/peri-menopausal women, and reduced odds of TNBC among post-menopausal women.

Previous studies have evaluated the association between measures of body composition and BC in various patient populations, with results suggesting a positive association of BMI with BC risk among post-menopausal women, and inverse association among pre-menopausal women [10–14]. This is consistent with our findings of significant inverse associations with BMI and weight for pre-menopausal women. A few studies have evaluated these associations among Nigerian women, although most were published over a decade ago prior to the accelerated increase in obesity rates in the region. Specifically, two separate studies observed that a higher waist-to-hip ratio was associated with significantly increased risk of BC [17, 18], and three studies showed that height was associated with significantly increased risk of BC [16, 17, 23]. Additionally, obesity (defined as BMI ≥ 30) was found to be associated with increased odds of BC, although the results were not statistically significant overall or among pre- or post-menopausal women [16].

For the first time, we present results on the association between measures of body composition and BC subtypes compared to controls among West African women. We found reduced odds of Luminal A, Luminal B, and HER2-enriched BC among pre/peri-menopausal women, and reduced odds of TNBC among post-menopausal women with increased BMI. Prior studies on this topic are conflicting, and suggest that the associations between body composition and BC subtypes vary by menopausal status. A case-control study among Black and White women in the United States documented a positive association between elevated waist-to-hip ratio and Luminal A BC among post-menopausal women [25]. Another analysis, which included studies in the Breast Cancer Association Consortium, a primarily European population, found that elevated BMI in younger women was more strongly associated with hormone receptor positive tumors (Luminal subtypes) [32], and another study from the United States noted that among post-menopausal women, BMI was associated with an increased odds of ER+/PR+ BC [33]. In contrast, a meta-analysis of studies from the US, Europe, and Asia found that ER+/PR+ tumors were less likely to develop among higher weight pre-menopausal women, but more likely to develop among higher weight post-menopausal women [34]. Our findings are consistent with a prior study that found an inverse association between BMI and risk of TNBC [35]. However, our findings are inconsistent with other past studies showing positive associations between obesity and TNBC [24, 25, 36], most of which were conducted in study populations from the United States, Europe, and Asia, and may not be representative of the BC phenotypes observed in Nigeria.

Despite the documented inverse association between BMI and BC risk among pre-menopausal women, the underlying biological mechanisms remain an active area of research. For instance, lower levels of progesterone among pre-menopausal obese women may lead to a lower risk of BC because progesterone is thought to have a primarily pro-proliferative role in the adult breast [37]. However, further research is needed to better explain how excess adiposity contributes to BC risk across different subtypes. While our findings may suggest lower BC risk among obese patients relative to lean patients, it is well-established that BC survival is negatively impacted by obesity [38]. These seemingly paradoxical observations, dubbed the obesity paradox, may be a consequence of differences in treatment response among differently weighted patients [39]. Additionally, biological mechanisms that explain the higher risk of TNBC in women of West African descent are not well-understood. We urge additional research among African populations to elucidate population-specific risk factors that may contribute to these outcomes.

Several considerations are relevant for our study. Given the predominance of the aggressive phenotype (high grade and TN subtype) of BC in Nigeria, also reflected in the current study population, it is possible for cases to have experienced disease associated weight loss prior to recruitment. It is worth noting that TNBCs grow quickly and are more likely to be clinically apparent compared with ER+/PR+ cancers, which are more likely to be detected by screening mammography [40]. The low prevalence of mammogram screening in Nigeria [41], reflected in our sample with less than 19% of cases having had a previous mammogram screening, may contribute to the high prevalence of TNBC. However, it is important to note that studies among African American women, with similar BC morphological features to Nigerian women [9], have been inconsistent, with some suggesting positive associations between excess adiposity and BC risk [42, 43], while others have reported no significant associations [44, 45]. The exact relationship between BMI and BC risk remains conflicting for women of African descent, indicating a need for further study in a larger cohort, especially given the context in which BC disease is distinctly more aggressive [6–8].

There are several strengths and limitations of this study relevant to the interpretation of these results. Since covariate data were self-reported at diagnosis, we cannot exclude the possibility of recall bias. However, our main exposures of interest, measures of excess adiposity, were obtained consistently across study participants by trained study personnel. Furthermore, because BMI, height, and weight were recorded at the time of diagnosis, we are unable to rule out the possibility of reverse causality. Additionally, we note the limitation of relying purely on BMI (height and weight) as a measure of excess adiposity. Individuals with the same BMI can have different body fat distributions [46]. Nevertheless, our study has important strengths, including the use of histologically confirmed cases of BC from multiple tertiary institutions in Nigeria where the majority of cancer patients receive treatment, and a vital contribution of diverse patient populations to the BC literature. Our study is the first to characterize the association between measures of body composition and BC risk among West African women by molecular subtype.

In conclusion, measures of body composition were associated with BC and these associations varied by molecular subtypes. Larger studies of women of African descent are needed to better characterize these associations and enhance population-specific BC prevention strategies.

## Data Availability

The datasets used and/or analyzed during the current study are available from the corresponding author on reasonable request.

## Abbreviations

BMI: Body mass index
BC: Breast cancer
CKD: Chronic kidney disease
ER: Estrogen receptor
HER2: Human epidermal growth factor-2
H3A: Human Heredity and Health Africa
IHC: Immunohistochemistry
MEND: Mechanisms for Established and Novel Risk Factors for Breast Cancer in Women of Nigerian Descent
PR: Progesterone receptor (PR)
SD: Standard deviation
TN: Triple-negative
